# Rad5, HLTF, and SHPRH: A Fresh View of an Old Story

**DOI:** 10.1016/j.tig.2018.04.006

**Published:** 2018-08

**Authors:** Menattallah Elserafy, Arwa A. Abugable, Reham Atteya, Sherif F. El-Khamisy

**Affiliations:** 1 Krebs Institute, Department of Molecular Biology and Biotechnology, Firth Court, University of Sheffield, Sheffield S10 2TN, UK; 2 Center for Genomics, Helmy Institute for Medical Sciences, Zewail City of Science and Technology, Giza 12578, Egypt; 3 These authors contributed equally to the manuscript

**Keywords:** RAD5, HLTF, SHPRH, cancer, HIV-1, bowel disease

## Abstract

Not only have helicase-like transcription factor (HLTF) and SNF2 histone-linker PHD-finger RING-finger helicase (SHPRH) proved to be important players in post-replication repair like their yeast counterpart, Rad5, but they are also involved in multiple biological functions and are associated with several human disorders. We provide here an updated view of their functions, associated diseases, and potential therapeutic approaches.

## *RAD5*, a Gene All Budding Yeast Laboratories Should Know about

Yeast has served as a prominent model organism in DNA repair research for decades [1]. Rad5 is a RING-finger-containing ubiquitin ligase and helicase that was discovered in budding yeast and was found to play a role in template switching (TS) and translesion synthesis (TLS) post-replication repair (PRR) mechanisms. TS utilizes a clever mechanism using the newly synthesized sister DNA strand as a template to bypass lesions, while TLS switches replicative polymerases to low-fidelity polymerases to overcome lesions [2].

The wild-type W303 yeast strain used by numerous laboratories harbors a *RAD5* mutation (*rad5-535*). Some researchers have started to use a strain in which the mutation has been corrected. Consequently, conflicting results have been reported in the literature by laboratories using the W303 *rad5-535* versus W303 *RAD5* strains [3]. While it is important to raise the awareness of yeast researchers to the importance of *RAD5* genotype in yeast, it is equally important to highlight how the mammalian Rad5 orthologs, such as HLTF and SHPRH, play multiple important roles in mammalian cells.

## HLTF and SHPRH; Rad5 Human Orthologs

**Figure 1:**
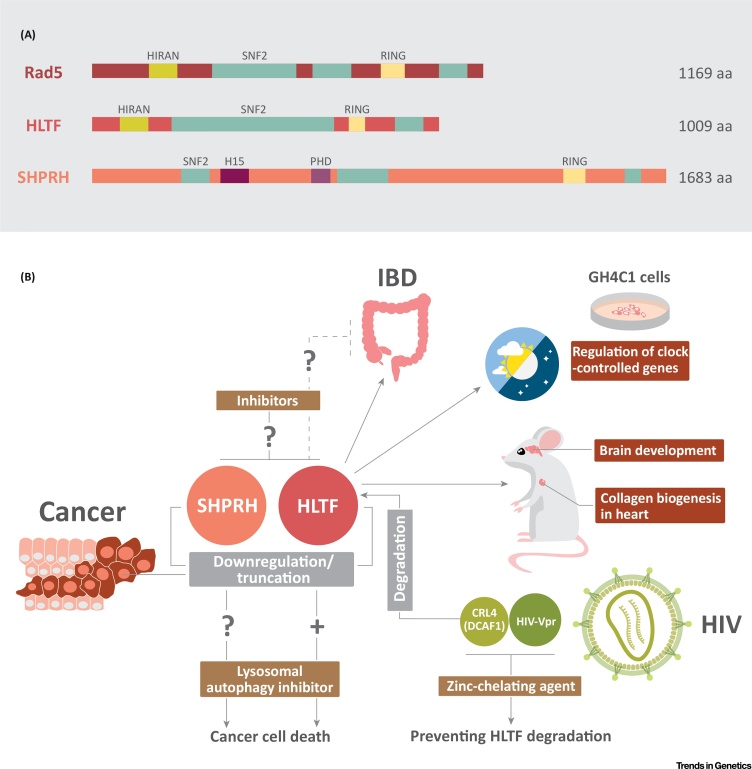
A Fresh View of HLTF and SHPRH. (A) Homology between Rad5, HLTF, and SHPRH. Similarly to Rad5, HLTF contains the conserved HIRAN domain. All three proteins contain the RING finger domain embedded between the SNF2 helicase motifs. SHPRH also contains a H15 linker histone H1/H5 domain and a PHD-finger domain. Panel adapted, with permission, from [2]. (B) Biological functions and associated diseases for HLTF and SHPRH, and potential therapeutic approaches. HLTF plays a role in the regulation of clock-controlled genes in GH4C1 cells, in mouse embryonic and postnatal brain development, and in collagen biogenesis in the heart. In addition, downregulation or truncation of HLTF and SHPRH have been implicated in different cancers. Cancer cells with low HLTF expression were found to be sensitive to lysosomal autophagy inhibitors. HLTF is also involved in REG1B overexpression, increasing the risk of colon cancer in IBD patients. Zinc-chelating agent prevents HLTF degradation by HIV-1. We propose testing the effect of the lysosomal autophagy inhibitors on cancers with low SHPRH levels. We also propose designing HLTF and SHPRH inhibitors to be tested in combination with lysosomal autophagy inhibitors. Abbreviations: aa, amino acids; IBD, inflammatory bowl disease.

HLTF is considered to be the closest ortholog to yeast Rad5; it shows higher sequence conservation to Rad5 than to SHPRH. HLTF contains the conserved HIRAN domain that is responsible for 3′-single-stranded (ss)DNA binding [4], and is able to complement UV sensitivity in the Δ*rad5* yeast strain. It also possesses double-stranded (ds)DNA translocase activity which is utilized to reverse stalled replication forks [5]. Similarly to yeast Rad5, both HLTF and SHPRH are E3 ubiquitin ligases and have been shown to polyubiquitinate PCNA, in cooperation with Ubc13–MMS2, in response to DNA damage, thereby directing PRR towards the TS repair pathway 6, 7. The homology between Rad5, HLTF, and SHPRH is depicted in Figure 1A [2].

## HLTF and SHPRH in Disease

### Cancer

A large body of evidence indicates that *HLTF* is a tumor-suppressor gene [2]. Methylation and silencing of *HLTF* have been documented in several cancers such as colon, gastric, esophageal, uterine, lung, hepatocellular, and bladder cancer 2, 8. *HLTF* promoter hypermethylation in the blood of cancer patients has been investigated as a tumor biomarker to predict prognosis and survival. Moreover, in cervical, thyroid, and head and neck cancers, alternative splicing of *HLTF* mRNA increases the expression of truncated versions lacking functional DNA repair domains [8]. Heterozygosity in the long arm of chromosome 6, where the *SHPRH* gene resides, has been reported in malignant melanoma, ovarian, and cervical cancers. Furthermore, *SHPRH* was found to be truncated or to contain missense mutations in tumor cell lines, which is consistent with a tumor-suppressor function [2].

Interestingly, *HLTF* was found to be one of the genetic determinants conferring sensitivity or resistance to lysosomal autophagy inhibitors such as hydroxychloroquine (HCQ) in several cancer cell lines [8]. HLTF expression was found to be low in HCQ-sensitive cell lines and high in resistant cell lines. HLTF protected against HCQ-induced cell death because it is essential for repairing the resulting reactive oxygen species (ROS)-induced DNA damage [8]. Because lysosome inhibitors are currently in clinical trials for efficacy as single or combined anticancer agents, HLTF expression profiling of patients may be helpful to determine the response [8] (Figure 1B). We suggest designing HLTF inhibitors for anticancer therapy because combining them with autophagy inhibitors could prove to be synergistic. Moreover, one could question whether cancers with downregulated SHPRH expression would also respond to autophagy inhibitors. Similarly, it is tempting to test whether inhibiting SHPRH would also synergize with autophagy inhibitors (Figure 1B).

### HLTF and HIV-1

Upon HIV-1 infection of target cells, viral protein R (Vpr) downregulates HLTF [9]. Vpr is known to recruit CRL4 (DCAF1) E3 ligase to mediate HLTF degradation, and thus facilitates viral replication. A highly conserved zinc-binding motif (HHCH) in Vpr is required for the interaction between Vpr and DCAF1 [9]. Mutations in this motif or the addition of a zinc-chelating agent, TPEN *N,N,N,N*-tetrakis-(2-pyridylmethyl) ethylenediamine, was found to disrupt the Vpr–CRL4 (DCAF1) interaction and therefore their function. Nevertheless, TPEN did not affect the assembly of the cellular CRL4 E3 complex. This opens the door to developing novel antiviral therapies against HIV-1 in which the interaction between Vpr and CRL4/DCAF1 is selectively disrupted [9] (Figure 1B).

### HLTF and Inflammatory Bowel Disease (IBD)

Gene expression analysis of regenerating gene (*REG*) family members has shown that the genes encoding REG-Iα (*REG1A*), REG-Iβ (*REG1B*), and REG-IV (*REG4*) are overexpressed in IBD. Upregulation of *REG* genes is beneficial on the one hand because it induces the proliferation of intestinal epithelial cells, protecting them from damage caused by the immune system, but on the other hand upregulation increases the risk of developing colon cancer [10].

A recent study investigating the link between *REG* genes and IBD reported that *REG1A*, *REG1B*, and *REG4* are overexpressed in the colon of Crohn’s disease patients, and that *REG4* is overexpressed in the colon of ulcerative colitis patients [10]. IL-22 was found to induce *REG1B* overexpression through activating the *REG1B* promoter region which contains a binding site for HLTF. Knockdown of HLTF was found to decrease the expression of IL-22-induced REG Iβ [10]. To prevent the development of colon cancer after intestinal epithelium recovery, the study suggested that anti-IL-22 and anti-REG proteins could be administered to patients. We also propose that developing inhibitors that prevent the interaction between HLTF and the *REG1B* promoter could be useful to reduce the expression of REG-1β after colon regeneration, and thus reduce the risk of colon cancer (Figure 1B).

## HLTF in Biological Functions

### HLTF in the Regulation of Clock-Controlled Genes (CCGs)

The majority of CCGs involved in circadian rhythm regulation lack the specific E-box response element required for regulation. A study was conducted on the GH4C1 rat pituitary somatolactotroph cell line to investigate the mechanism of transcription of prolactin (*Prl*), a CCG that lacks the specific E-box element. HLTF was found to interact with PIT-1 transcription factor at the P2 region of the *Prl* promoter, which is different from the specific E-box that is usually bound by CCGs. HLTF and PIT-1 are not rhythmically expressed; however, HLTF interacts with NONO and SFPQ proteins which rhythmically bind to the *Prl* gene. Therefore, this leads to *Prl* expression that is necessary for maintaining the normal circadian pattern. These findings have uncovered a novel role for HLTF in the regulation of the oscillatory expression of CCGs that lack the specific E-box response element [11] (Figure 1B).

### HLTF in Mouse Embryonic and Postnatal Development

HLTF has been shown to play crucial roles in embryonic and postnatal development of the murine heart and brain 12, 13. In mouse heart, HLTF is necessary for hypoxia-inducible factor 1α (HIF-1α) regulation to achieve normal collagen biogenesis. In addition, HLTF regulates the expression of the cohesion and condensin complexes, as well as of cell-cycle regulators, during embryonic development of mouse brain. Therefore, a significant percentage of HLTF null mice die, and those that survive suffer from DNA damage, increased apoptosis in brain, brain encephalomalacia, G2/M transition defects, and fibrillar network disorganization 12, 13 (Figure 1B).

## Concluding Remarks

There is increasing interest among scientists in discovering different roles for the Rad5 human homologs. HLTF is especially interesting because any associated defects would not only affect the DNA repair activity in cells but also the expression level of multiple genes that are potentially implicated in different disorders. We propose that defects in HLTF and SHPRH could be associated with multiple disorders that have not yet been identified. They could also be utilized as biomarkers and exploited as potential therapeutic targets. Further work will shed light on novel approaches to selectively target the perturbed functions of HLTF and SHPRH while retaining their important role in unaffected tissues.
